# Supplementary figures and images for: Environmental and Sensor Integration Influences on Temperature Measurements by Rotary-Wing Unmanned Aircraft Systems
Source: Sensors (Basel). 2019 Mar 26;19(6):1470. doi: 10.3390/s19061470 (PMC6471934; doi:10.3390/s19061470)

# Flight 2 - Wind Estimator Off

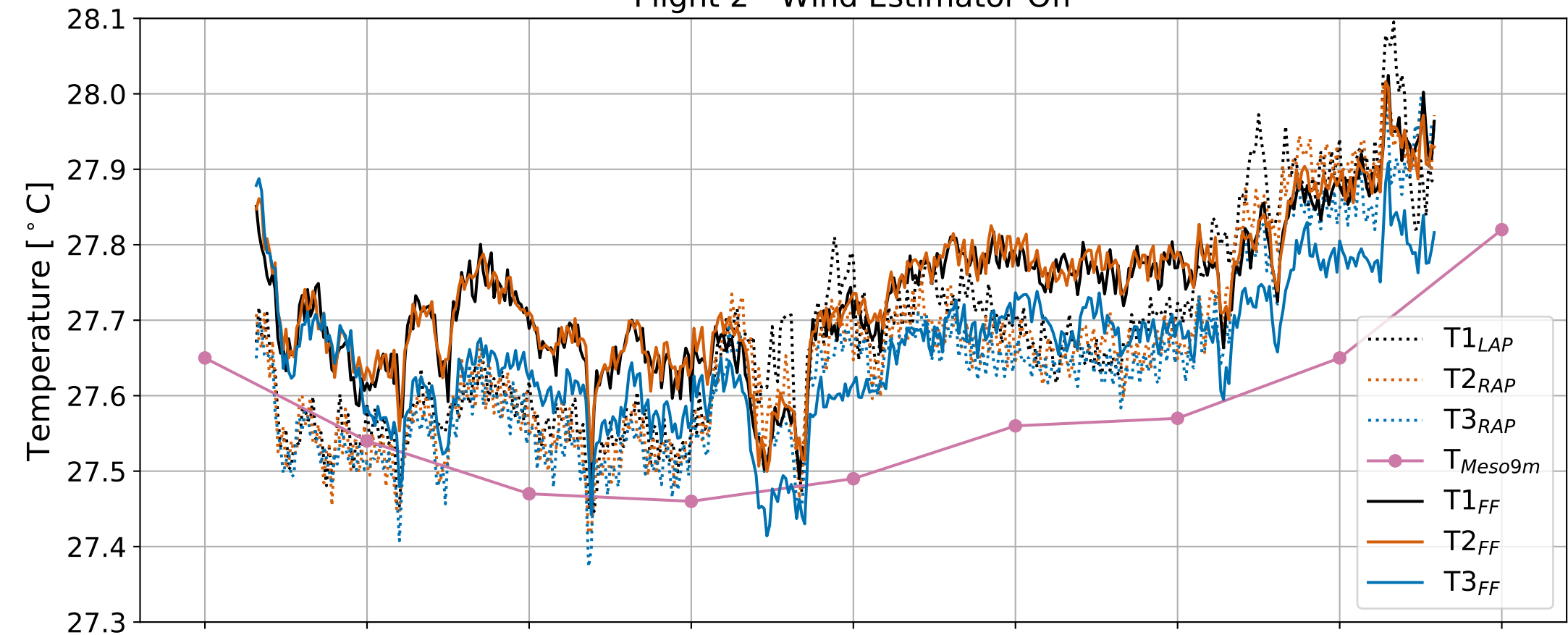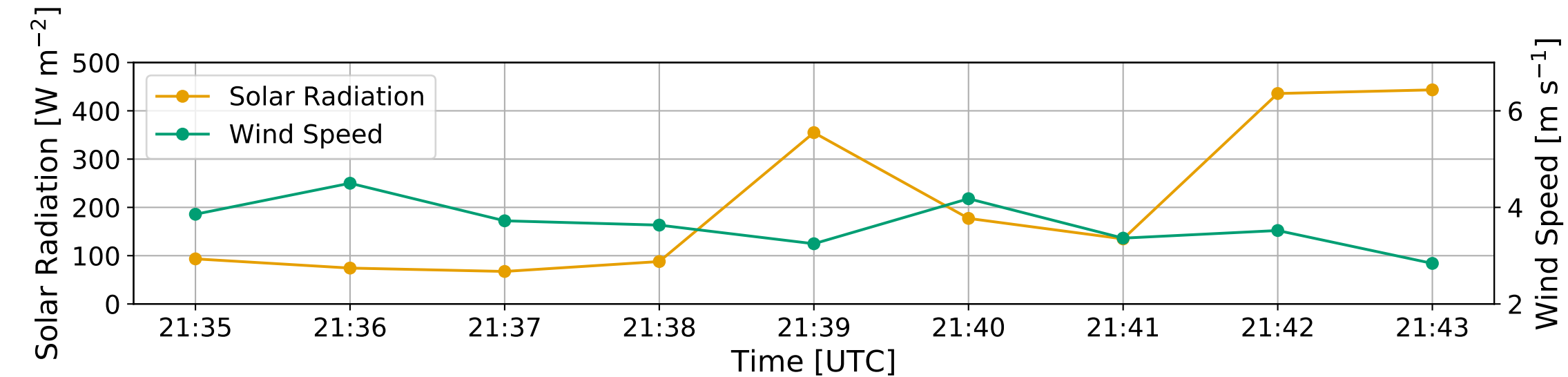

Supplement: Supplementary file 1 [file sensors-19-01470-s001.zip › Supplementary/SFig01.pdf]

Flight 3 - Wind Estimator On

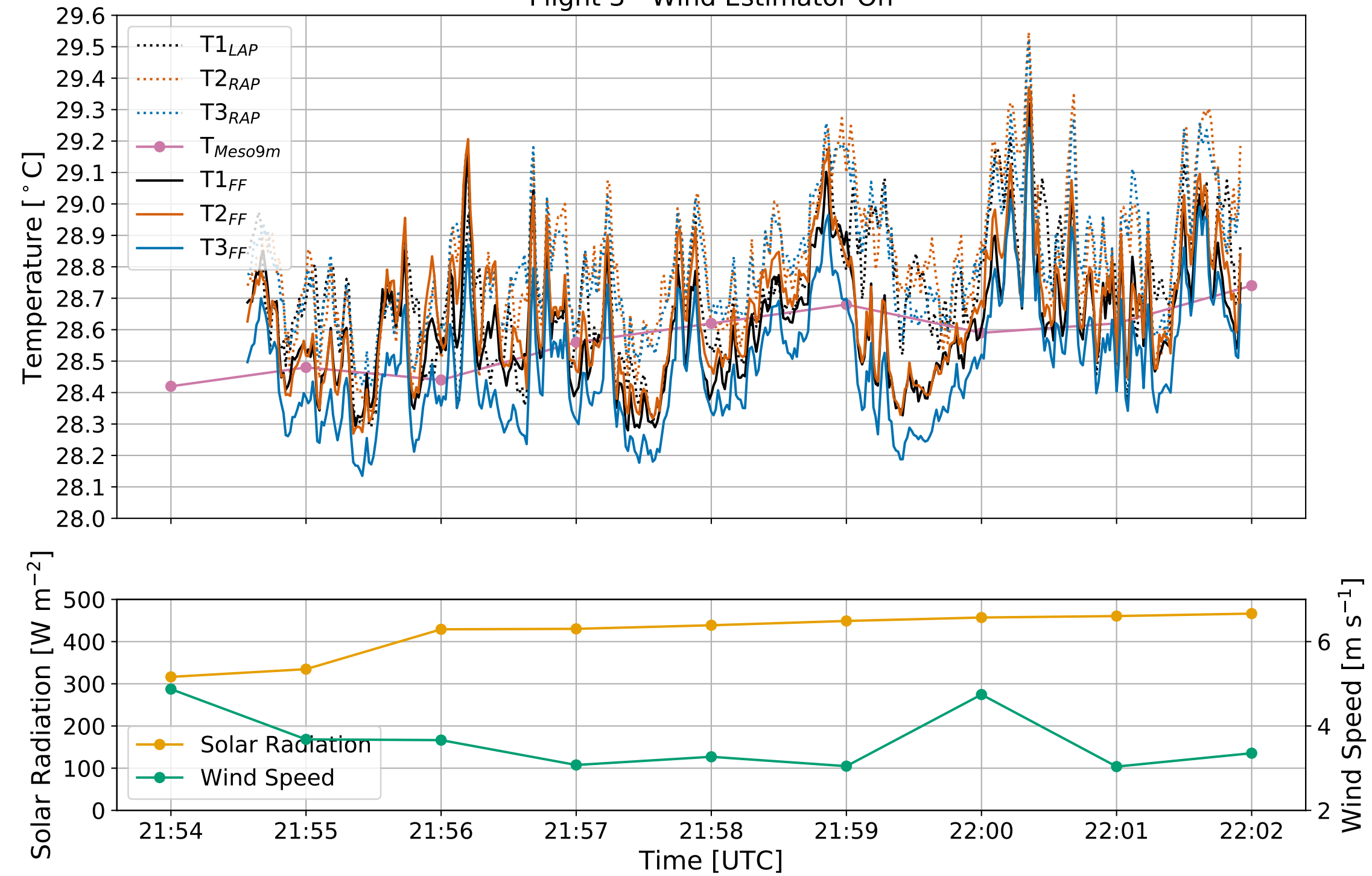

Supplement: Supplementary file 1 [file sensors-19-01470-s001.zip › Supplementary/SFig02.pdf]
